# Assessment of Admissions to Psychiatric Intensive Care Unit (PICU) at Farnham Road Hospital, Guildford: A Clinical Audit

**DOI:** 10.1192/bjo.2024.633

**Published:** 2024-08-01

**Authors:** Hardeep Singh, Waseem Sultan, Karim Alame, Reuben Tagoe

**Affiliations:** Surrey and Borders NHS Foundation Trust, Surrey, United Kingdom

## Abstract

**Aims:**

The primary aim was to analyze three months of admissions to Rowan Ward PICU (February 22 to April 2022) according to NAPICU's 2014 criteria, followed by implementing recommendations and conducting a re-audit (November 2022 to January 2023) to assess their impact. Secondary objectives included examining the link between prior PICU admissions and higher readmission rates, even when not clinically necessary.

**Methods:**

Methods involved assessing each admission against NAPICU's criteria and reviewing the reason for admission (RFA) for appropriateness. Data collection utilized various sources, including SystmOne, Mental Health Act assessments, and referral documents. Collaborative analysis with the PICU consultant was conducted due to the subjective nature of RFA interpretation.

**Results:**

Results from the initial audit revealed that 12 out of 36 patients (33%) were deemed unsuitable for PICU admission, with 8 having prior PICU admissions (67%). Only 22% had documented multidisciplinary team (MDT) discussions. In the subsequent audit, 9 out of 38 patients (24%) were deemed unsuitable for PICU admission, with 2 having prior admissions (22%). Only 3% had documented MDT discussions.

**Conclusion:**

There was a reduction in inappropriate admissions from 33% to 24% in the subsequent cycle. This improvement was linked to the implementation of recommendations from the first audit, such as introducing a standardized referral form, enhancing consultant-to-consultant communications, and forming a PICU outreach team. While the initial findings indicated higher readmission rates for patients with prior PICU admissions, this trend lessened in the subsequent evaluation. However, there is still insufficient documentation of Multidisciplinary Team (MDT) discussions, highlighting the need for a re-audit to accurately assess any changes.

